# Are Immigrants and Nationals Born to Immigrants at Higher Risk for Delayed or No Lifetime Breast and Cervical Cancer Screening? The Results from a Population-Based Survey in Paris Metropolitan Area in 2010

**DOI:** 10.1371/journal.pone.0087046

**Published:** 2014-01-22

**Authors:** Claire Rondet, Annabelle Lapostolle, Marion Soler, Francesca Grillo, Isabelle Parizot, Pierre Chauvin

**Affiliations:** 1 Perre Louis Institute of Epidemiology and Public Health, Department of social epidemiology, INSERM, Paris, France; 2 Pierre Louis Institute of Epidemiology and Public Health, Department of social epidemiology, Sorbonne Universités, UPMC Univ Paris 06, Paris, France; 3 Department of General Practice, School of Medicine, UPMC Univ Paris 06, Paris, France; 4 Centre Maurice Halbwachs, Research Team on Social Inequalities, CNRS, Paris, France; Vanderbilt University, United States of America

## Abstract

**Objectives:**

This study aims to compare breast cancer screening (BCS) and cervical cancer screening (CCS) practices of French women born to French parents with those of immigrants and nationals born to immigrants, taking their socioeconomic status into account.

**Methods:**

The study is based on data collected in 2010 in the Paris metropolitan area among a representative sample of 3000 French-speaking adults. For women with no history of breast or cervical cancer, multivariate logistic regressions and structural equation models were used to investigate the factors associated with never having undergone BCS or CCS.

**Results:**

We confirmed the existence of a strong gradient, with respect to migration origin, for delaying or never having undergone BCS or CCS. Thus, being a foreign immigrant or being French of immigrant parentage were risk factors for delayed and no lifetime screening. Interestingly, we found that this gradient persisted (at least partially) after adjusting for the women’s socioeconomic characteristics. Only the level of income seemed to play a mediating role, but only partially. We observed differences between BCS and CCS which suggest that organized CCS could be effective in reducing socioeconomic and/or ethnic inequities.

**Conclusion:**

Socioeconomic status partially explained the screening nonparticipation on the part of French women of immigrant origin and foreign immigrants. This was more so the case with CCS than with BCS, which suggests that organized prevention programs might reduce social inequalities.

## Introduction

In France, breast cancer is the most common cancer in women, with an incidence of 53,000 new cases in 2011, and cervical cancer ranks twelfth, with 2810 new cases in that year. In this country, screening tests are recommended for these two female cancers. Breast cancer screening (BCS) is done either through the national BCS program, in which screening is proposed to eligible 50- to 74-year old women every other year, or as an individual, opt-in screening procedure [1], [2]. France’s organized screening program has been in operation since 2004. Previously, only individual, opt-in BCS was available to women. In 1988, before there was an organized program, 10.3% of women aged 55 to 64 were screened annually, and in the early 1990s, 3 million mammograms were already being performed in France every year in women of all ages [3]. The use of the Pap smear has become widespread since the 1970s, and the French guidelines, which target women 25 to 65 years of age, recommend that they undergo cervical cancer screening (CCS) every 3 years after two normal Pap smears one year apart [4], [5]. The nonparticipation of vulnerable women in breast and cervical cancer screening is largely described in France [1], [4], [5], where it has been shown that having a low education level, being unemployed and having a low monthly household income are risk factors for being overdue for such screening. The existence of a strong gradient in screening practices according to immigration status has been reported in New Zealand and the United States [6]–[9] but little is known about the situation of immigrant women in France. Indeed, since available data on immigration are usually scarce in French health surveys and information systems, CCS is the only type of female cancer screening that has been studied in this connection [4], and no study has ever compared access to breast cancer screening with that to cervical cancer screening on the basis of immigration status in France. The objectives of our study were to determine the prevalence of delayed and no lifetime screening among French women of immigrant origin and among foreign immigrants, and to estimate and compare the associations between immigration status and either delayed or no lifetime BCS or delayed or no lifetime CCS among women living in the Paris metropolitan area. We also sought to test how women’s socioeconomic status (SES) could have a mediating effect on the association between their immigration status and their screening practices.

## Materials and Methods

### Study Sample and Outcomes

The SIRS (a French acronym for “Health, Inequalities and Social Ruptures”) survey was conducted in the winter of 2009/2010 among a representative sample of the adult French-speaking population in the Paris metropolitan area for the purpose of studying social inequalities in health and in access to health care. The sample consisted of 3006 adults aged 18 to 101 years. It employed a stratified, multistage cluster sampling procedure that overrepresented the poorer neighborhoods (census blocks). Its design, methods and sample representativeness have been reported previously [4], [5], [10]. A questionnaire with a large number of sociodemographic and health-related questions was administrated face-to-face during home visits. In this survey, the variables of interest were delayed and no lifetime BCS and CCS, as self-reported by the women.

### Cervical Cancer Screening

The Papanicolaou (Pap) smear is the main screening modality for early detection and improved chances of survival from cervical cancer [11]. In France, since 1995, it is recommended that a Pap smear be performed every 3 years after two normal annual smears [12]. We therefore decided to use a 3-year threshold to divide the adult female population into two subpopulations for the analyses of delayed CCS (3 years or less since their last smear test, or more than 3 years). In order to study no lifetime cervical cancer screening, we once again divided the adult female population into two subpopulations: those who had never been screened for cervical cancer and those who had been screened for such cancer at least once during their lifetime (regardless of the frequency). In the SIRS survey, the date of the last screening test was self-reported by the women.

### Breast Cancer Screening

The mammogram is a screening procedure for the early detection of breast cancer. In France, since 2004, it is recommended that a mammogram be performed every 2 years between the ages of 50 and 74 years. For our delay analyses, we decided to use a 2-year threshold to divide the female population into two subpopulations (2 years or less since their last mammogram, or more than 2 years). In order to study no lifetime breast cancer screening, we divided the female population into two subpopulations: those who had never had a mammogram and those who had had at least one mammogram during their lifetime (regardless of when it or they were performed). As with CCS, the date of the last screening test was self-reported by the women.

### Survey Populations

For this survey, we considered four groups of women because of the different ages for having each type of screening.

To study delayed CCS, we considered women aged 25 to 65 (in line with the French recommendations). To study never-screening for cervical cancer, we considered all women over the age of 25. Women who had had a hysterectomy were excluded from the analysis in both groups.

To study delayed BCS, we considered women aged 50 to 74, and to study no lifetime mammography, we considered all women over the age of 50. Indeed, even though organized BCS in France is recommended only for women aged 50 to 74, we decided to look at women over the age of 80, since they were in the target group when the BCS recommendations were widely disseminated. Women who had had breast cancer were excluded from the analysis in both groups.

### Independent Variables

As we usually did in a number of previous analyses [4], [5], [13], the women’s origin was divided into the following categories: French women born to two French parents (whom we will refer to as “women of French origin” in the rest of this paper), French women born to at least one foreign parent (French women of immigrant origin) and women of foreign nationality (foreign immigrants). This variable was labeled “immigration status” in the rest of the text.

As for the respondents’ socioeconomic status (SES), we considered their education level, monthly household income per consumption unit (in four categories based on the distribution quartiles in the study sample), and employment status (in four categories: working, unemployed, at home, and students and retired women grouped together).

### Statistical Methods

Because of a significant difference in the age distributions between the foreign immigrants and the French women of immigrant origin and women of French origin (the first two groups were younger than the women of French origin), we decided to calculate age-standardized rates for each screening test using 2008 national census data for the Paris metropolitan area. The comparisons between proportions were tested using the Pearson chi-squared test. Logistic regression models were used, first to estimate the age-adjusted association between immigration status and delayed and no lifetime screening, and then to estimate it by further taking the women’s SES into account. All the regression models were estimated specifying that the collected data were clustered by census block. A p-value <0.05 was considered significant for all the statistical analyses presented.

Lastly, in order to test the mediating effect of SES on the relationship between immigration status and screening practices, we used structural equation modeling [14] to estimate the direct and indirect SES-mediated effects of immigration status on screening practices (for BCS and CCS successively) and to calculate the proportion of the total effect that might be due to SES. Initially, SES was introduced into a path analysis as a latent variable consisting of the combination of the three SES characteristics (education level, household income and employment status). The root mean square error of approximation (RMSEA) and the comparative fit index (CFI) were used to assess the models’ fit. All the analyses were performed with STATA 12 software.

Ethics statement: This cohort study received legal authorization from two French national authorities for non-biomedical research: the *Comité consultatif sur le traitement de l’information en matière de recherche dans le domaine de la santé* (CCTIRS) and the *Commission nationale de l’informatique et des libertés* (CNIL). The participants provide their verbal informed consent. Written consent was not necessary because this survey did not fall into the category of biomedical research (as defined by French law) and did not collect any personal identification data.

## Results

### Description of the Survey Populations

The final SIRS sample consisted of 3006 persons, 1819 of whom were women. Of them, 27 were excluded from the analyses of delayed CCS because they had had a hysterectomy. Therefore, the sample for studying delayed CCS consisted of 1347 women (aged 25–65 years). The sample for studying no lifetime CCS consisted of 1724 women (aged 25–98 years). Of the 1819 women in the SIRS sample, 85 were excluded from the analysis of BCS because they had had breast cancer. Therefore, the sample for studying delayed BCS consisted of 614 women (aged 50–74 years), and the sample for studying no lifetime BCS consisted of 783 women (aged 50–98 years).

### Standardized Prevalence of Delayed and no Lifetime CCS According to Immigration Status

**Figure 1:**
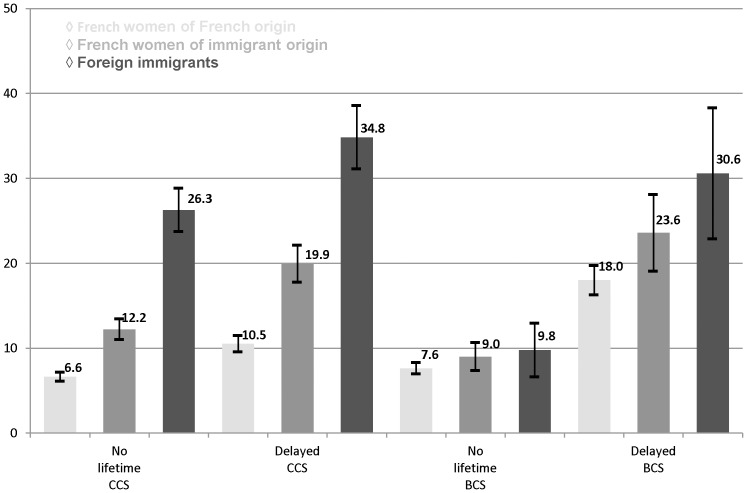
Standardized prevalence of delayed and no lifetime screening according to the women’s immigration status.

As shown in Figure 1, there was a gradient according to immigration status for the four outcomes. This gradient was steeper for cervical cancer screening than for breast cancer screening. Indeed, 10.5% (95% CI = [9.6–11.5]) of the women of French origin, 19.9% (95% CI = [17.8–22.1]) of the French women of immigrant origin and 34.8% (95% CI = [31.1–38.6]) of the foreign immigrants (p<0.001) had delayed their CCS. As well, 6.6% (95% CI = [6.1–7.2] of the women of French origin reported no lifetime CCS, while 12.2% (95% CI = [11.0–13.4]) of the French women of immigrant origin and 26.3% (95% CI = [23.7–28.8]) of the foreign immigrants (p<0.001) did so.

### Characteristics of CCS According to Immigration Status

Table 1 shows the results of the age-adjusted bivariate and multivariate linear regression models for delayed and no lifetime CCS. The association with the women’s immigration status was significant for both outcomes. Thus, the foreign immigrants were at significantly greater risk than the French women of immigrant origin of never having undergone CCS, and the latter, in turn, were at significantly greater risk of this than the women of French origin. The risk of being overdue for CCS was also higher in the foreign immigrants (OR = 3.52 [2.40–5.17]) and the French women of immigrant origin (OR = 1.76 [1.27–2.44]) than in the women of French origin. Introducing socioeconomic variables showed that having a low education level and a low monthly household income were also significantly associated with both outcomes. Introducing these variables into the model caused the estimate of the strength of the association with immigration status to decrease only partially.

**Table 1:** Association between immigration status and delayed and no lifetime cervical cancer screening in women aged 25–44 years in the Paris metropolitan area, 2010: adjusted for age, then further estimated by taking the women’s socioeconomic status into account.

		Never-screeners							Overdue screeners					
	Total n*	%	*p*	OR [95% CI]	*p*	OR [95% CI]	*p*	Total n*	%	*p*	OR [95% CI]	*p*	OR [95% CI]	*p*
***Age***														
25–44	695	10.2	<0.001	Ref.	<0.001	Ref.	<0.001	695	16.3	0.398	Ref.	0.746	Ref.	0.622
45–64	679	5.0		0.59 [0.38–0.90]		0.48 [0.30–0.78]		652	14.6		1.06 [0.75–1.48]		0.90 [0.60–1.36]	
≥65	350	13.4		2.05 [1.34–3.15]		1.23 [0.62–2.45]		–	–	–			–	
***Immigration*** ***status***														
Woman ofFrench origin	1168	5.7	<0.001	Ref.	<0.001	Ref.	<0.001	863	11.1	<0.001	Ref.	<0.001	Ref.	0.003
French woman ofimmigrant origin	318	11.8		2.24 [1.53–3.27]		1.83 [1.29–2.60]		288	18.1		1.76 [1.27–2.44]		1.24 [0.90–1.71]	
Foreignimmigrant	218	21.1		4.47 [2.86–6.97]		3.07 [1.92–4.90]		196	30.6		3.52 [2.40–5.17]		2.13 [1.38–3.29]	
***Education*** ***level***														
Tertiary	801	4.2	<0.001			Ref.	<0.001	675	9.5	<0.001			Ref.	0.001
Secondary	756	10.2				1.78 [1.21–2.83]		582	19.1				1.47 [1.05–2.05]	
None orprimary	167	24.6				3.77 [2.19–6.49]		90	36.7				2.50 [1.35–4.60]	
***Monthly household*** ***income (per CU)***														
≤1115.83 €	487	16.8	<0.001			Ref.	0.036	402	26.9	<0.001			Ref.	0.002
>1115.83 €but ≤1733.33	463	7.6				0.61 [0.39–0.95]		369	14.9				0.66 [0.46–0.95]	
>1733.33 €but ≤2605 €	412	4.9				0.50 [0.30–0.83]		312	9.0				0.44 [0.26–0.74]	
>2605 €	362	4.1				0.51 [0.25–1.06]		264	6.4				0.32 [0.14–0.71]	
***Socio-occupational*** ***group***														
Working	918	5.8	<0.001			Ref.	0.407	894	12.3	<0.001			Ref.	0.108
Unemployed	97	9.3				0.86 [0.36–2.05]		97	18.6				0.95 [0.46–1.96]	
At home	189	16.4				1.41 [0.78–2.52]		173	24.3				1.16 [0.73–1.86]	
Apprentice, studentor retired	508	11.6				1.38 [0.72–2.66]		171	21.8				1.67 [1.09–2.66]	

* The number for each dependent variable category for which the prevalence of overdue and never-screeners is given.

**Figure 2:**
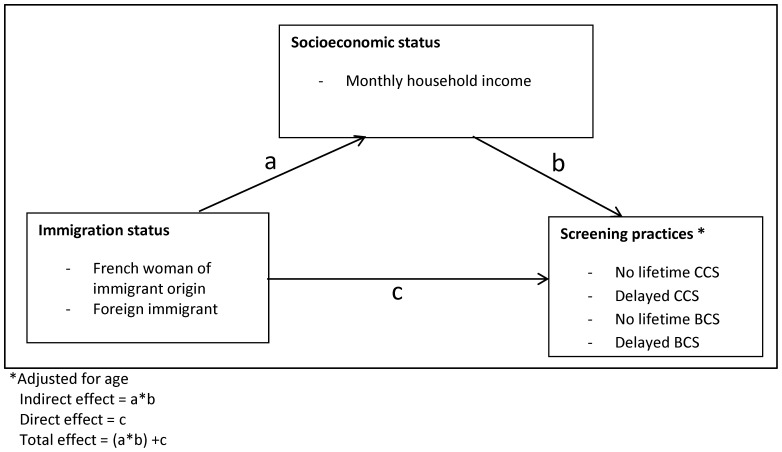
Structural equation model. Mediating model of the association between immigration status and delayed or no lifetime screening.

In a structural equation model, we initially modeled socioeconomic status as a latent variable, but this model was not well fitted to the data (for both outcomes, the RMSEA and CFI were not within the acceptable range). Consequently, we decided to individually test the socioeconomic variables as mediators. Only the introduction of monthly household income led to a well-fitted model (shown schematically in Figure 2, the RMSEA and CFI being given in Table 2). In the case of no lifetime CCS, we observed that the proportion of the effect of immigration status mediated by monthly household income was 31.1% for the French women of immigrant origin and 16.6% for the foreign immigrants (Table 2). For delayed CCS, these proportions were, respectively, 60.3% and 25.8%. Overall, the models’ fit appears to be quite good, since the RMSEA was lower than 0.08 and the CFI greater than 0.90 for CFI.

**Table 2:** Results of the structural equation models.

	Cervical cancer screening				Breast cancer screening			
	No lifetime	p	Delayed	p	No lifetime	p	Delayed	p
*** French-born to at least one foreign parent***								
Indirect effect	0.021	<0.001	0.044	<0.001	0.008	0.04	0.012	0.14
Direct effect	0.045	0.01	0.029	0.25	0.005	0.85	0.044	0.35
Total effect	0.066	<0.001	0.072	0.003	0.012	0.60	0.056	0.05
**Mediated portion**	**31.1%**		**60.3%**		**50.4%**		**21.0%**	
*** Foreigners***								
Indirect effect	0.027	<0.001	0.052	<0.001	0.014	0.03	0.020	0.13
Direct effect	0.135	<0.001	0.147	0.03	0.060	0.07	0.104	0.09
Total effect	0.162	<0.001	0.199	<0.001	0.080	0.02	0.124	0.06
**Mediated portion**	**16.6%**		**25.8%**		**16.7%**		**16.8%**	
RMSEA^1^	0.06		0.08		0.02		0.04	
CFI^2^	0.953		0.963		0.994		0.980	

Measure of the mediating effect of monthly household income on the association between immigration status and delayed and no lifetime screening.

1 Root mean square error of approximation.

2 Comparative fit index.

### Standardized Prevalence of Delayed or no Lifetime BCS According to Immigration Status

We observed the same gradient for BCS in relation to immigration status as for CCS. Thus, 18.0% (95% CI = [16.3–19.7] of the French women had delayed BCS, while this was the case with 23.6% (95% CI = [19.1–28.1]) of the women of immigrant origin and 30.6% (95% CI = [22.9–38.3]) of the foreign immigrants, but the difference was not significant. As for no lifetime BCS, only 7.6% (95% CI = [6.9–8.3]) of the French women reported never having undergone BCS compared to 9.8% (95% CI = [6.6–12.9]) of the foreign immigrants. Here, too, this difference was not significant.

### Characteristics of BCS According to Immigration Status

Unlike for CCS, only foreign immigrants seemed to be at higher risk for being breast cancer never-screeners (table 3, OR = 2.23; 95% CI = [1.02–4.87]), but this result should not be taken at face value because the overall estimate was not significant (p = 0.125). The other ORs were not significant, and, in fact, their punctual estimates were notably lower than those calculated for CCS (in a different population and with different age limits). Here, too, introducing socioeconomic variables partially decreased the respective OR estimates. All of them became non-significant (including an OR close to 1 for the French women of immigrant origin and no lifetime BCS). In multivariate analysis, neither immigration status nor socioeconomic status was significantly associated with delayed or no lifetime BCS. In a structural equation model, it is noteworthy that monthly household income accounted for 50.4% of the total effect of immigration status on no lifetime BCS in the French women of immigrant origin and for 16.7% of that effect in the foreign immigrants (Table 2). In the case of delayed BCS, these proportions were estimated to be 16.8% and 21%, respectively.

**Table 3:** Association between immigration status and delayed and no lifetime breast cancer screening in women aged 50 and over in the Paris metropolitan area, 2010: adjusted for age, then further estimate by taking the women’s socioeconomic status into account.

		Never-screeners							Overdue screeners					
	Total n*	%	*p*	OR [95% CI]	*p*	OR [95% CI]	*p*	Total n*	%	*p*	OR [95% CI]	*p*	OR [95% CI]	*p*
***Age***														
50–59	306	4.3	<0.001	Ref.	<0.001	Ref.	<0.001	306	18.7	0.362	Ref.	0.206	Ref.	0.566
60–74	308	3.3		0.83 [0.31–2.25]		0.50 [0.21–1.20]		308	21.6		1.28 [0.88–1.82]		1.17 [0.69–1.99]	
≥75	169	16.6		5.04 [2.45–10.37]		2.56 [1.13–5.81]			–	–			–	
***Immigration*** ***status***														
Woman ofFrench origin	604	5.8	0.164	Ref.	0.125	Ref.	0.379	472	18.4	0.098	Ref.	0.171	Ref.	0.444
French woman ofimmigrant origin	121	7.4		1.31 [0.63–2.69]		1.05 [0.47–2.35]		91	23.9		1.39 [0.74–2.60]		1.24 [0.63–2.46]	
Foreignimmigrant	58	12.1		2.23 [1.02–4.87]		1.76 [0.79–3.92]		51	30.0		1.90 [0.97–3.72]		1.56 [0.79–3.11]	
***Education*** ***level***														
Tertiary	117	3.8	0.003			Ref.	0.417	213	20.5	0.1932			Ref.	0.219
Secondary	347	6.9				1.33 [0.57–3.06]		210	17.7				0.72 [0.49–1.05]	
None orprimary	319	12.8				1.82 [0.72–4.61]		60	26.8				0.98 [0.51–1.86]	
***Monthly household*** ***income (per CU)***														
≤1115.83 €	184	12.0	0.008			Ref.	0.323	145	28.2	0.046			Ref.	0.168
>1115.83 € but≤1733.33 €	190	4.7				0.49 [0.20–1.19]		155	16.3				0.57 [0.31–1.04]	
>1733.33 € but≤2605 €	202	4.5				0.45 [0.17–1.20]		154	19.7				0.71 [0.41–1.24]	
>2605 €	207	5.3				0.63 [0.25–1.61]		160	17.1				0.53 [0.29–0.98]	
***Socio-occupational*** ***group***														
Working	274	2.2	0.004			Ref.	0.344	272	16.5	0.190			Ref.	0.819
Unemployed	24	8.3				2.27 [0.23–22.95]		24	27.3				1.33 [0.52–3.39]	
At home	54	7.4				2.32 [0.51–10.52]		49	27.1				1.31 [0.59–2.91]	
Apprentice, studentor retired	426	9.2				2.78 [0.90–8.58]		264	21.7				1.20 [0.70–2.06]	

* The number for each dependent variable category for which the prevalence of overdue and never screeners is given.

## Discussion

Our study sought to describe the role of immigration status in women’s cancer screening practices among a representative sample of French-speaking adults in the Paris metropolitan area. For CCS, we found that 8.8% of the women had never undergone CCS during their lifetime and that 15.5% were overdue, with noticeable differences according to their immigration status. Indeed, together with the overall figure for widespread cervical cancer screening practices, which is consistent with that reported in previous French studies [15], our study outlines certain demographic (immigration-related) and socioeconomic inequalities. Being a foreigner or of immigrant origin was a risk factor for being an overdue screener or a never-screener, as was observed in previous studies [5], [16]. Interestingly, we found that this gradient persisted (at least partially) after adjusting for the women’s socioeconomic characteristics.

Regarding BCS, 6.5% of the women reported never having had a mammogram during their lifetime, and 20.2% were overdue for this examination. These findings are consistent with those of other French studies [1], [17], in which fewer than 10% of the women had never had a mammogram. They are also comparable to findings in other countries, such as Sweden, where this proportion was 5.6% in 1990 [18], and the United States, where it was 11% in 2003 [19]. Although our study did not find any significant differences in these proportions according to immigration status, in bivariate analysis, being of foreign nationality was associated with a significantly higher risk of being a never-screener.

A low education level and a low monthly household income are two SES characteristics widely described as being associated with participation in BCS [18]–[20] and/or CCS [1], [21]–[23] in the literature. We have shown in this survey that, in the Paris metropolitan area, after adjustment for immigration status, they are both still associated with the risk of being an overdue screener or a never-screener for cervical cancer but not for breast cancer.

Of course, our results are limited by our sample size and the statistical power of our analysis, since there were only 51 breast cancer never-screeners and 122 overdue screeners. Also, only French-speaking women had been interviewed in the SIRS survey, and since language could be a barrier to accessing health care, the differences between foreign immigrants and French women could have been even greater if non-French speaking women had been included in the study. In addition, our immigration status variable did not detail the foreign nationalities. Even though this information was available in our dataset, the numbers were too small to perform our analysis by nationality. Such specific studies are necessary because it is known that there are large disparities between minority groups [6]. Finally, since all our data are declarative, all our results may be tainted by classification or desirability biases. However, upon examining the substantial strengths of the estimated associations, it is reasonable for us to believe that our results are meaningful and that they cannot be completely explained by such biases.

Certain findings are worth noting with regard to the mediating effect of SES on the association between immigration status and women’s participation in cancer screening as estimated by our structural equation models.

First, the level of income seemed to play a mediating role for both breast and cervical cancer screening in a context where BCS mammograms are free of charge in France and Pap smear tests are mostly covered by France’s social security health insurance. This may be due to the facts that some complementary examinations for BCS (e.g., breast ultrasound) may not be fully covered by public health insurance and that, although low, the total out-of-pocket cost of CCS (approximately 14 €) may not be insignificant for the poorest women.

On the other hand, this mediating effect of the level of income is only partial, which suggests that other mediators may play a role, apart the material and financial ones, for instance, a lack of information, low health literacy and/or the persistence of body- and health-related norms. These norms can differ between immigrant groups according to the prevailing norms in the cultures or countries of origin of recent arrivals [5]. Explanations such as less access to health-care services, including prevention [6], [24], and certain psychosocial variables (social support, social network, cancer fatalism and breast cancer worry) should be considered as well [6], [7]. Some authors suggest that screening participation might also be modulated by body image. For instance, obese and/or overweight women may be significantly more likely to delay CCS or BCS [25], it being known that obesity is more prevalent in certain minority groups and in low-income groups, generally in developed Western countries [26], including in the Paris metropolitan area [27].

Finally, the overall gradient between the women of French origin, the French women of immigrant origin and the foreign immigrants can be explained by the women’s acculturation, which keeps French women born to immigrants in an intermediate position between foreign immigrants and women of French origin [28].

We reported certain differences in social gradients between BCS and CCS. Thus, immigration status and socioeconomic variables were significant risk factors for both CCS outcomes, but not for BCS. This could be due to the fact that organized screening programs may help reduce “ethnic” and/or socioeconomic disparities by offering a systematic (and free) examination to all the women of the target ages, regardless of their social status, and by using specific strategies to reach the most underserved women (what some authors refer to as “proportional universalism strategies” [29]) when opportunistic screening, such as CCS, may not. In France, most, but not all, women are followed by a medical gynecologist. Those who are not are followed, at best, by general practitioners (GPs), only 51% of whom are reported to do Pap smears themselves [30]. In our survey, 29.5% of the women reported that they were not being followed regularly for their gynecological health (either by a specialist or a GP), and this proportion increased as one goes down the social ladder.

Many interventions have been proposed to reduce disparities in women’s cancer screening, and the results are often discordant [28], [31]. Nonetheless, a recent meta-analysis showed that some of them might increase CCS participation among ethnic minority women [32]. For example, the authors found that access-enhancing strategies and community education are effective in improving Pap test use among such women. Of the proposed access-enhancing strategies, financial incentives (i.e., reductions in payment and direct compensation to patients) were the strongest patient-targeted intervention approach. This suggests that well-organized CCS might be effective in reducing social inequalities.

In conclusion, our study indicates that more specific strategies targeting foreign immigrants and, more broadly, socially vulnerable women need to be implemented to reduce inequalities in women’s cancer screening. Not only can an organized CCS program reduce these inequalities, but even within the framework of organized programs, immigrant women and French women of immigrant origin need to avail themselves of specific measures in order to reach levels similar to those observed in the majority population in terms of practices and participation.
